# Acoustic Event Detection in Vehicles: A Multi-Label Classification Approach

**DOI:** 10.3390/s25082591

**Published:** 2025-04-19

**Authors:** Anaswara Antony, Wolfgang Theimer, Giovanni Grossetti, Christoph M. Friedrich

**Affiliations:** 1 Department of Computer Science, University of Applied Sciences and Arts (FH Dortmund), 44227 Dortmund, Germany; anaswara.antony005@stud.fh-dortmund.de; 2 Volkswagen Infotainment GmbH, 44803 Bochum, Germany

**Keywords:** acoustic event detection, audio scene, autonomous driving, transformers

## Abstract

Autonomous driving technologies for environmental perception are mostly based on visual cues obtained from sensors like cameras, RADAR, or LiDAR. They capture the environment as if seen through “human eyes”. If this visual information is complemented with auditory information, thereby also providing “ears”, driverless cars can become more reliable and safer. In this paper, an Acoustic Event Detection model is presented that can detect various acoustic events in an automotive context along with their time of occurrence to create an audio scene description. The proposed detection methodology uses the pre-trained network Bidirectional Encoder representation from Audio Transformers (BEATs) and a single-layer neural network trained on the database of real audio recordings collected from different cars. The performance of the model is evaluated for different parameters and datasets. The segment-based results for a duration of 1 s show that the model performs well for 11 sound classes with a mean accuracy of 0.93 and F1-Score of 0.39 for a confidence threshold of 0.5. The threshold-independent metric mAP has a value of 0.77. The model also performs well for sound mixtures containing two overlapping events with mean accuracy, F1-Score, and mAP equal to 0.89, 0.42, and 0.658, respectively.

## 1. Introduction

Everyday sounds, such as honking and sirens, are a few examples that assist a driver to make split-second decisions, which are crucial to one’s safety. The automotive soundscape is rich with diverse sounds that not only help the driver to be alert but also make sense of the surroundings. Similarly, autonomous vehicles can also benefit from these acoustic cues for safer self-driving. Currently, autonomous driving technologies are predominantly based on data acquired from visual sensors, such as cameras and long- and short-range sensors like RADAR and LiDAR [1]. Reports from car manufacturers also suggest that autonomous vehicles rely on these vision sensors to sense surroundings with only a few exceptions, like Waymo from Google [2]. The Hearing Car project from Fraunhofer IDMT (https://www.idmt.fraunhofer.de/en/institute/projects-products/projects/the_hearing_car.html, accessed on 2 January 2024), Embedded siren detection from Bosch (https://www.bosch.com/stories/embedded-siren-detection/, accessed on 2 January 2024), I-SPOT project by EU MSCA [3] are among the few recent approaches that equip future cars with a sense of hearing. Academic research on autonomous driving technologies follows a similar pattern. Thus, when it comes to sense the surroundings, the cars are able to “see” the environment but still lack the capability “to hear”. Research has shown that compared to other sensory signals, sound is less sensitive to harsh weather conditions and occlusion of objects, which helps in better environmental perception [4]. This also provides additional safety and better navigation possibilities. If used to their full potential, audio sensors can thus complement existing sensor modalities by providing backup in the event of a subsystem failure and reducing uncertainty [2]. Acoustic Event Detection (AED) is a step forward in this direction.

**Figure 1:**
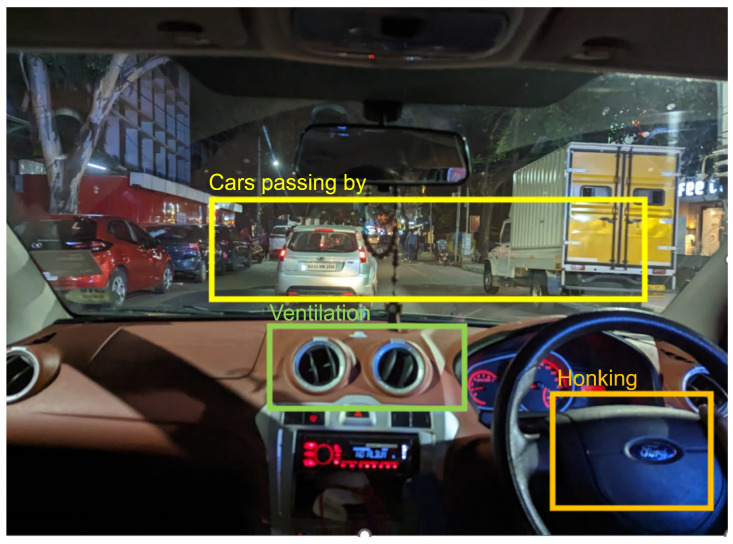
Example of an auditory scene that can be perceived inside a car. Inside, one can hear the sound of the ventilation, engine, honking, passing cars, etc. These can be identified as separate sound events that may overlap each other or may not overlap each other. The colored boxes on the figure represent some of these sources of sounds.

**Figure 2:**
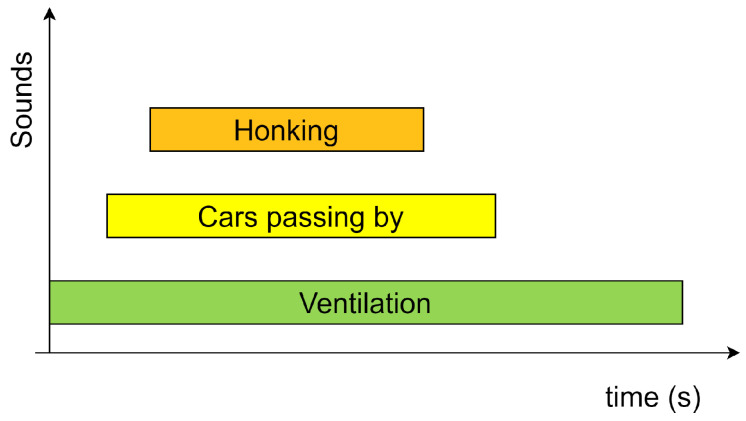
Acoustic Event Detection of the audio scene. The sound of ventilation, cars passing and honking, along with the onset and offset times of these events, are detected. The length of the rectangle is proportional to the duration of each sound and thus marks the start and end times. The rectangles are placed one above the other to indicate overlapping time intervals.

AED, also known as the Sound Event Detection (SED) system, detects different sound events in an input audio along with the temporal instances at which they occur [5]. Depending on the extent of the overlap of events, an AED system can be monophonic or polyphonic. A monophonic AED system refers to a system that does not detect overlapping events, while a polyphonic system can detect overlapping events [6]. Polyphonic AED is more challenging compared to monophonic AED since distinguishing the features of individual sounds is difficult. Natural acoustic environments are polyphonic in nature, implying the existence of multiple sounds at a certain point in time with varying amplitude, timbre, pitch, etc. The paper focuses on developing a polyphonic AED system that can detect the various overlapping sound events within a car. Consider an auditory scene that can happen inside a car as shown in Figure 1. The goal of the proposed AED system is to detect these events along with the time of occurrence as described in Figure 2. Predicting the activity of multiple sound classes for short audio segments can produce such an audio scene description. This implies that the AED problem can be seen as a multi-label classification problem on short audio segments. If the system provides predictions for the duration of the whole audio, it is referred to as tagging, while if the predictions describe the activity pattern of the audio classes, it is referred to as detection [7].

### Related Work

During early research in AED, machine learning techniques that make use of SVM, HMM, GMM, and NMF were used. Although non-neural network-based methods do not require large amounts of strongly labeled data, they do not perform very well on polyphonic AED tasks [6]. With the advancements in computing resources, DNN with many linear layers that can jointly learn to extract acoustic features and classify them started to emerge. Other architectures, such as FNN, CNN, and BLSTM were also explored to solve AED problems. These architectures were followed by hybrid models with more than one network architecture. One of the most popular hybrid models used in AED is CRNN [6]. The architecture consists of CNN layers stacked with RNN layers that have specific functions [8]. Architectures, such as BLSTM [9] and bidirectional GRU [10], constitute the RNN layers. One of the drawbacks of neural network-based approaches is that they require a large amount of training data [6]. Using approaches like deep transfer learning, the limitation of acquiring a large amount of data can be solved [11]. With the advent of Transformer architecture [12], many approaches using pre-trained transformer models started to emerge [13,14]. From the literature on AED systems, it can also be inferred that they are mostly focused on environmental sounds and everyday sounds that are freely available from audio datasets such as AudioSet [15], ESC50 [16], Freesound [17], etc. The number of sound samples that originate in a vehicle environment is limited in these datasets. The paper introduces an AED system that is trained on a dataset containing the sounds in an automotive context.

Key contributions of this paper include the following:

- An ontology that introduces a shared vocabulary to set a context for collecting audio data exclusive to the automotive environment. To the best of our knowledge, an ontology for sound data in an automotive context has never been proposed.
- Data augmentation methodology to generate synthetic polyphonic sound mixtures with integrated user and command-line interfaces. The methodology can create polyphonic sound mixtures representing audio scenes that are close to reality. The method proves to be efficient for generating synthetic training data.
- An Acoustic Event Detection model for vehicles that is trained using actual recordings from cars.

The following section introduces the proposed ontology, data augmentation methodology and describes how the AED system is developed using a multi-label classification approach. It gives an overview of the proposed AED system and the components involved. The performance of the classification model under various influencing factors is summarized in the results section.

## 2. Materials and Methods

**Figure 3:**
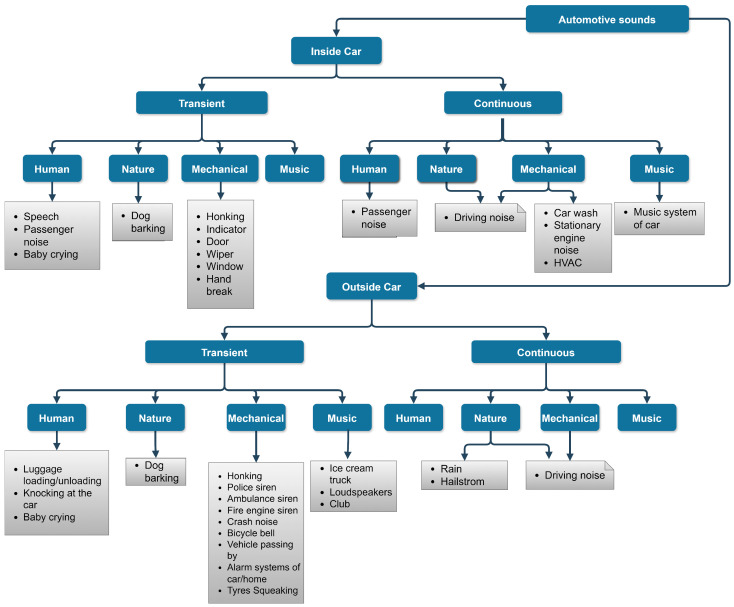
Ontology for automotive sounds. The coloured rectangles represent high-level semantics: ‘Inside Car’ and ‘Outside Car’ to indicate where the sounds originate with respect to the vehicle. They are further classified on the basis of their transient/continuous nature and the sources. The solid white rectangle represents the ‘labels’ with which each sound is identified. Some of the sounds are best described with more than two groups of sound sources, represented in a rectangle with an earmark.

The first step in any sound event classification/detection task is to establish a context to collect audio data that reflect sounds in an automotive environment. Existing ontology, such as the one prescribed in the AudioSet dataset [15] covers a wide range of sound events. However, a deeper look into the ontology shows that they are more generic and do not cover some of the sounds that are exclusive to the automotive environment. For instance, the sound of wipers, indicators, HVAC, etc. Therefore, an ontology described in Figure 3 proposes a hierarchical categorization of different sounds that originate in an automotive environment. The ‘Urbansound Dataset 8K’, which derived the taxonomy for urban sounds, defined the four top-level groups for sound sources as human, nature, mechanical and music [18]. Similar top-level groups can also be applied in this case. Note that the ontology is a work in progress and can be reformulated later by adding or removing events.

### 2.1. Methodology

The performance of multi-label classification models heavily depends on the complexity of the model as well as the amount of training data available. The freely available audio datasets contain a limited number of sound samples from the defined ontology. Therefore, deep transfer learning approaches using pre-trained models that are trained in large audio data can be used for downstream classification tasks [11]. This approach does not require a large amount of data in the domain task, which, in this case, is the classification of the sounds as mentioned in the ontology. There are many freely available pre-trained networks that are trained on large audio datasets. They are mostly based on the CNN [19] and Transformer [12] architectures.

The performance of these networks on the AudioSet dataset, the complexity of the model in terms of the number of parameters and information on the license are summarized in Table 1. It can be inferred from the table that the transformer-based network, BEATs [20] shows high performance on the AudioSet dataset, as well as the highest complexity compared to others. In addition, the license does not restrict its free usage. The complexity of the model cannot be ignored, as training such large models requires sufficient resources. However, the pre-trained model weights of BEATs are freely available and can be used as a frozen feature extractor. By doing so, the model is not retrained, retaining the same weights. Moreover, BEATs uses a self-supervised learning approach during pre-training, and therefore, the representation learning network of BEATs will not show bias towards labels in the source domain task. BEATs was also used as a frozen feature extractor in the baseline for the DCASE 2023 challenge on Sound Event Detection with Weak Labels and Synthetic Soundscapes (https://dcase.community/challenge2023/task-sound-event-detection-with-weak-labels-and-synthetic-soundscapes, accessed on 19 February 2024). All these factors make BEATs a good candidate. For resource-constrained environments, models such as YAMNet that offer good performance with much less complexity can be used. BYOL also offers good performance in downstream classification tasks compared to YAMNet with few training parameters; however, the value of mAP on Audio Set is not available and, therefore, is not considered.

**Figure 4:**
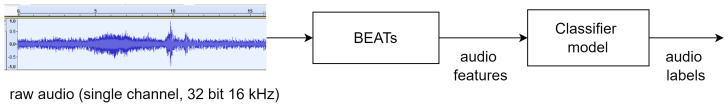
Proposed approach for the AED system.

Figure 4 summarizes the approach proposed for the AED system using the pre-trained network BEATs [20]. Single channel, 32-bit audio with a sampling rate equal to 16 kHZ is fed to the BEATs representation network, which will extract features of the input audio (refer to Section 2.5). These audio features are fed to the downstream classifier model, which represents the additional layers on which the target domain data are trained. Due to the high complexity of the BEATs model, its model parameters can be kept frozen. This means that during training, only the parameters of the classifier model will be updated to perform multilabel classification. Thus, BEATs acts as a frozen feature extractor while the classifier model is trained on these features to predict the corresponding labels.

**Figure 5:**
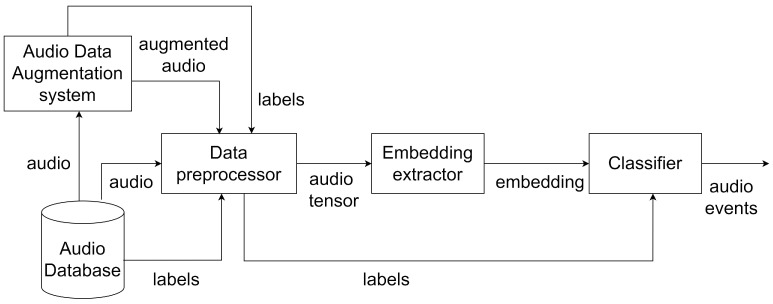
Overview of AED system. The audio data from the Audio Database are fed to the Audio Data Augmentation system for data augmentation. Data preprocessor converts the input audio along with the augmented audio into the tensor format [25] to convert them into embeddings by the embedding extractor. The Classifier model is trained using these embeddings and the corresponding labels.

Figure 5 describes the structure of the proposed AED system during the training phase. The different parts of the system are as follows:

- Audio Database: Provides audio data and the corresponding labels with the onset and offset times to train and test the classifier
- Audio Data Augmentation system: Responsible for generating augmented audio with overlapping sound events and its corresponding labels
- Data preprocessor: Processes audio data and labels for the embedding extractor and classifier, respectively.
- Embedding extractor: Transforms the input audio into embeddings that encapsulate the audio features
- Classifier: Learns to predict the audio events from these embeddings using the reference labels provided by the data preprocessor

### 2.2. Audio Database

The Audio Database corresponds to the audio data for training, evaluating, and testing the classifier model. Audio events mentioned in the ontology are recorded using three A2B microphones [26] fitted on the roof of the car. Manufactured by Analog Devices, each A2B chip (node) is a digital microphone module consisting of three microphone channels. It uses MEMS technology for the microphone modules with analog to digital conversion integrated into the AD242x chip. Using the microphones, recordings were collected from three different Volkswagen cars driving around the city of Bochum. The recorded audio (48 kHz, 9 channel, wav file format) is manually annotated to generate label files (csv file format), which contain information on the sound events along with their onset and offset times.

There are also a few datasets that contain some of the audio events mentioned in the ontology, which are freely available on the Internet. To increase the amount of audio data, data were also collected from these datasets. The audio database thus corresponds to audio data from the two sources:

1. Audio recordings using A2B microphones: This corresponds to 71 min of audio recordings of 14 different sound events; Baby crying, Dog barking, Door, Driving noise, HVAC, Honking, Indicator, Knocking at the car, Passenger noise, Speech, Stationary engine noise, Vehicle passing by, Window, Wiper, available in both flac and wav formats.
2. Internet: Audio data corresponding to the events in the ontology that are downloaded from Pixabay (https://pixabay.com/sound-effects/, accessed on 24 February 2024). They are mainly available in MP3 and wav file formats. More data can be collected from the Internet on the basis of the results of the experiments using the database from the recordings.

#### Exploratory Data Analysis (EDA)

**Figure 6:**
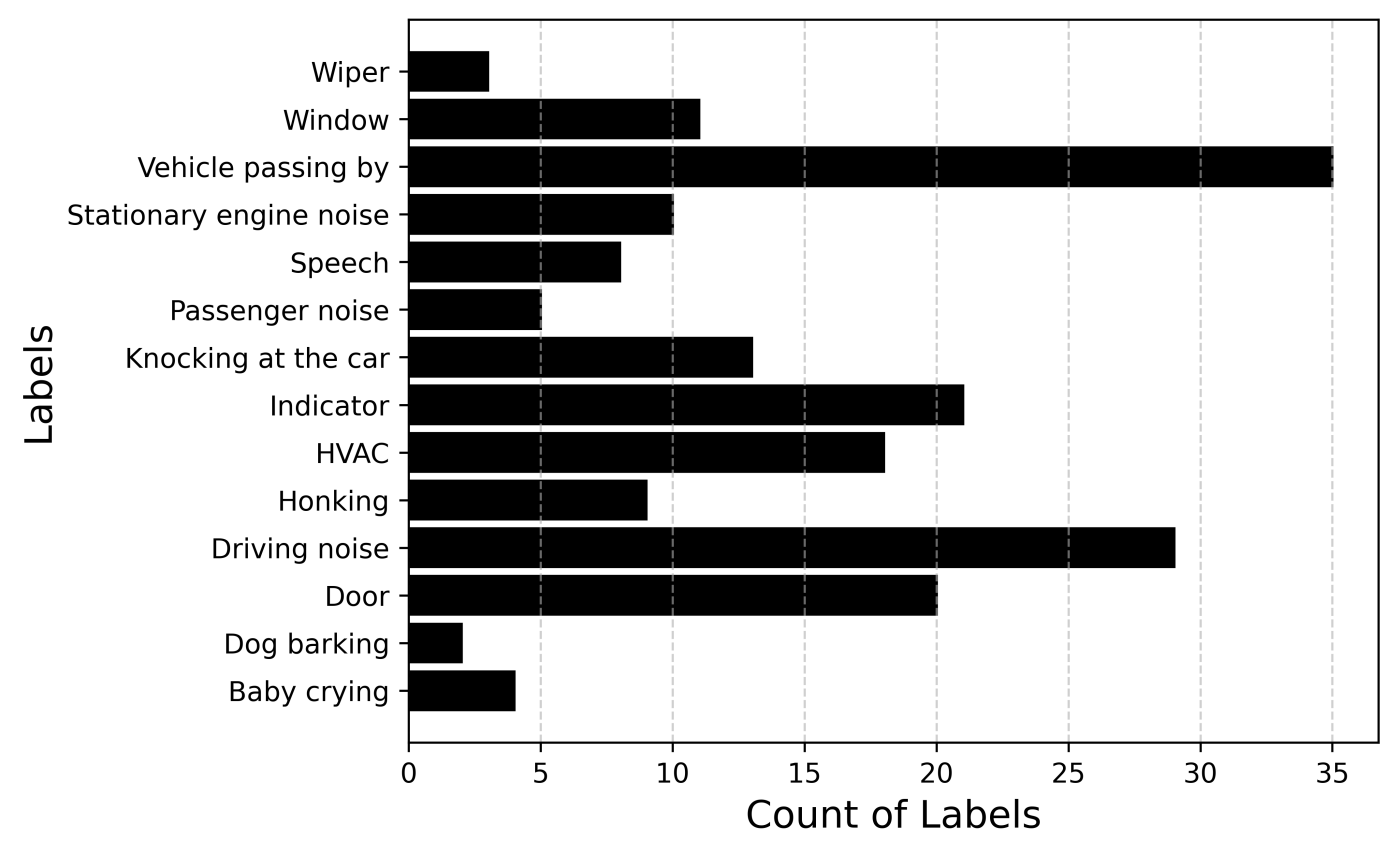
Total number of samples available for each audio event.

**Figure 7:**
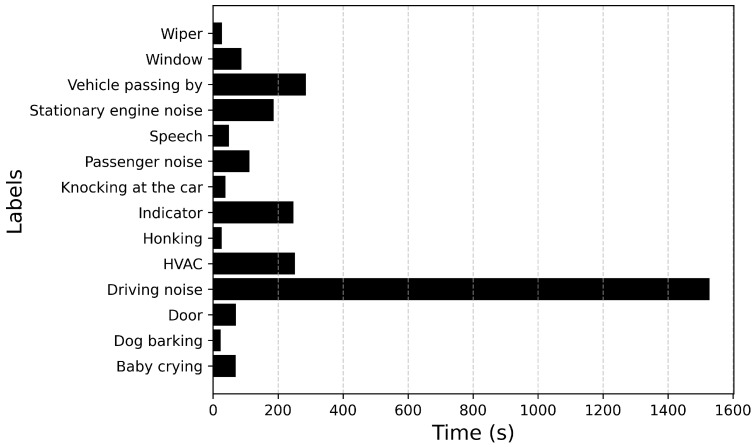
Total duration of each recorded audio event.

Figure 6 and Figure 7 describe the audio database containing recordings of 14 events totaling up to 71 min of audio data. Figure 6 plots the number of audio recordings for each label. It must be noted that these recordings contain only the annotated label and there is no overlapping of events. It can be inferred from the figure that the dataset is heavily imbalanced with a large number of samples available for events such as Driving noise and Vehicle passing by while only a few samples are available for events such as Baby crying and Wiper. If the imbalance value is defined as the highest number of samples (Vehicle passing by: 35) to the lowest number of samples (Dog barking: 2), then the imbalance ratio of this dataset will be 17.5.

Figure 7 plots the total duration of each recorded sound event. From Figure 7 it can be inferred that the event Driving noise has the longest duration. This could be attributed to the nature of the sound. Events such as Door, Honking are transient in nature, whereas events such as Stationary engine noise, HVAC, Driving noise are continuous in nature. The duration of the sound events also contributes to the imbalance in the dataset. After analyzing the duration of each sound event annotated across all recordings, the maximum duration value is for the event Driving noise with a duration of 148 s, while Honking has the lowest duration of 538 ms.

### 2.3. Audio Data Augmentation System

In order to increase the data for training and testing the model, an audio augmentation methodology is proposed that considers different augmentation techniques. The component ADA in Figure 5 corresponds to the system that makes use of this augmentation methodology to generate augmented audio by mixing individual sound events. This can be used to generate synthetic training data containing multiple overlapping sound events leading to polyphonic sound mixtures. This approach is also good for accurate annotations, as the start and end times are determined by the mixing algorithms [7]. They can also be used to simulate a variety of acoustic situations or auditory scenes. Therefore, the system helps to generate data for polyphonic AED. The proposed augmentation methodology is described below:

Let $\left\{s_{i}\left(t\right)\right\}$ be the set of sound signals to be mixed and *i* be the sound signal index where i=1,2,…,I and n=0,1,2,…,N be the time index.

Let ${\hat{s}}_{i}\left(n\right)$ be the normalized sound signal, which can be defined as

(1) $${\hat{s}}_{i}\left(n\right)=\frac{s_{i}\left(n\right)}{max(s_{i}\left(n\right))}$$

**Figure 8:**
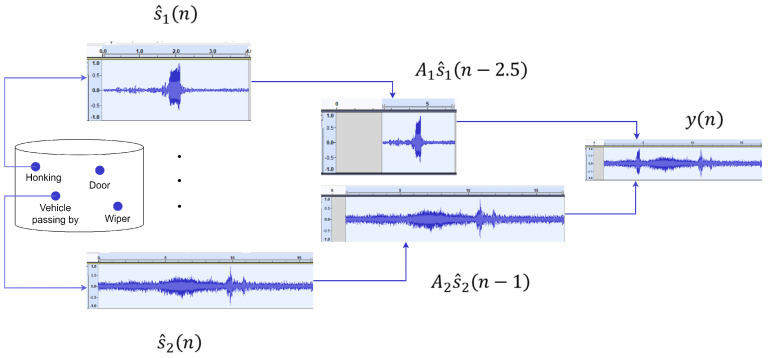
Augmentation overview. The labels ‘Honking’ and ‘Vehicle passing by’ are selected from the database. The normalized signals are scaled by a random value in the range [0, 1] with the start of the signals time shifted by values chosen randomly from the user-specified range for each signal. The final augmented audio y(n) contains the sound of vehicles passing by and honking with the applied time shifts and amplitude scaling.

Thus, the range of the amplitude values of the normalized signal will be in the interval [−1, 1]. This helps to achieve a uniform range of amplitude values for all signals. Now, the mixing equation can be defined as

(2) $$y\left(n\right)=\sum_{i=1}^{I}A_{i}\cdot {\hat{s}}_{i}\left(n-k_{i}\right)\;\forall i=1,2,\ldots ,I$$

where $A_{i}$ represents the amplitude scaling factor, which can be any random value from the interval [0, 1] and $k_{i}$ is a random time shift selected from the range specified by the user corresponding to each sound signal. Figure 8 gives an overview of the augmentation process. As can be seen from the figure, the user can select one or more sound events, apply amplitude scaling in a selected range, and mix them within a specified range of time-shift. The user can select any number of sound events as well as control the extent of overlapping of sound events by changing the range of time-shift. In this way, ADA can be used to generate a large amount of synthetic data for training, testing and evaluation.

### 2.4. Data Preprocessor

The data preprocessor is the component for processing the audio data, as well as the labels (annotations) from the Audio database and the ADA system for training and testing the classifier. It is mainly responsible for the following;

1. Processing of labels
2. Processing of audio
3. Generating datasets for iterative-stratified validation

**Figure 9:**
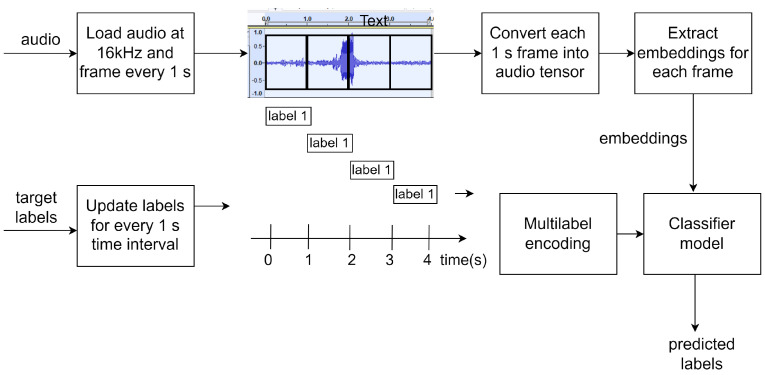
Overview of data processing for classifier. The input audio signal must be filtered and resampled to a sampling frequency of 16 kHz since BEATs accepts audio sampled at this frequency. A window of 1 s duration is applied on the input audio to obtain frames of length 1 s. These frames are converted to tensor format and fed into the BEATs model to generate embeddings for each frame. The annotations must also be updated for every 1 s duration. In the multi-label encoding stage, they are converted to a binary matrix that contains the presence (1) or absence (0) of each class/label for each 1 s frame. The classifier is trained to classify the embeddings corresponding to one second of input audio from these reference labels in a supervised learning format.

Multi-label classification on short temporal segments of audio can describe the activity of the selected sound classes in an audio. In order to define the temporal resolution for prediction, examining the statistics of the training data or general assumptions about the target events is recommended [7]. BEATs uses a window length of 25 ms with an overlap of 10 ms, resulting in audio frames of 25 ms duration. The minimum duration of the audio event in the training data is 573 ms, while the median duration is up to 6.75 s. Since 25 ms is too short based on the annotated duration of each audio event and most of the annotated events have at least a duration of one second, a duration of one second seems to be a more reasonable choice for the temporal resolution. Pre-trained networks such as YAMNet also have a prediction interval of 0.96 s that approximates 1 s [21]. Figure 9 describes how audio and labels are processed by the data preprocessor so that for a given input audio, the classifier predicts audio events every 1 s.

#### Iterative Stratification

One of the drawbacks of cross-validation is that it splits the dataset into disjointed sets of equal size [27]. If the dataset is unbalanced with a large variation in the number of samples available for each class, then a stratified version of the cross-validation method is used, where the distribution of classes across each fold is maintained. Iterative stratification is an alternative for the stratification of multi-label data. The algorithm is particularly effective when the dataset has rare class samples [27]. As can be inferred from Section Exploratory Data Analysis (EDA), the audio database is heavily imbalanced with an imbalance value of 17.5. Therefore, to split the dataset for multilabel classification, iterative stratification is used. The data preprocessor is also responsible for generating these partitions of the dataset based on iterative stratification. It makes sure that the same recordings are not split into the training and validation sets. The number of folds proposed for iterative stratification is 10.

### 2.5. Embedding Extractor

The component Embedding extractor generates embeddings that represent features of a given input audio. According to the proposed approach for the AED system, the embedding extractor in this case is the BEATs model. The model accepts input audio in tensor format [25] and extracts embeddings that represent its features from the input audio. These embeddings can be the input features for the downstream task [7]. In this case, they are the input features for the classifier to be trained.

The backbone network of BEATs uses a Vision Transformer (ViT) [28] structure with a Linear Projection layer and a stack of Transformer Encoder layers. The backbone network consists of 12 Transformer Encoder layers, 768-dimensional hidden states, and 8 attention heads. One of the most commonly used audio features in Transformer-based networks is the Mel filter bank features [13,29,30]. Given an input audio waveform of t seconds, BEATs extracts the 128-dimensional Mel filter bank features by applying a sliding Povey window [31] of 25 ms every 10 ms. This will result in a 128×100t spectrogram that is normalized to a mean of 0 and a standard deviation of 0.5 [20]. The spectrogram is split into *N* patches of size 16×16 with an overlap of 6 in both the time and frequency dimensions. This input sequence of *N* patches, where N=12[(100t−16)/10] is fed to the Linear Projection Layer, where each 16×16 patch is flattened to a 1D patch embedding of size 768 by the Linear Projection Layer. To capture the spatial structure of the 2D audio spectrogram, a trainable positional embedding of size 768 is also added [23]. The resulting sequence is fed to the Transformer Encoder layers to obtain an embedding of dimension 768.

### 2.6. Classifier

The BEATs model uses a task-specific linear classifier on the encoder layer to generate labels for downstream classification tasks [20]. Following a similar approach, a linear classifier is considered for the classifier model of the AED system. The BEATs model shows good results for downstream tasks such as audio classification and speech classification [20]. A linear classifier can be a neural network or algorithms such as SVM.

#### 2.6.1. Linear Classifier

The linear classifier receives the input embeddings and calculates the output probabilities of each class/label. Classes with a probability higher than the defined threshold are predicted classes. Since BEATs generates embedding of dimension 768, the input vector to the classifier network will be a tensor of dimension 768 utilizing the full representation network, while the output vector will be of dimension 14 corresponding to the number of labels. Thus, the proposed classifier model is a single-layer neural network with 768 input neurons and 14 output neurons. A single layer is chosen, as adding layers would increase complexity and can affect real-time inference capabilities.

When an audio segment of a duration of 1 s is fed to the BEATs model, it extracts 128 dimensional features every 10 ms, generating 100 frames with a dimension equal to 128 (100, 128). These features have to be converted to patches of size 16×16. After confirming with one of the authors of the BEATs paper, it is understood that since 100 frames are not divisible by 16, it will discard the last 4 frames to obtain 96 frames. They are later converted to 16×16 patches to obtain 48 patches ((128/16)·(96/16)). This is fed to the backbone network with 768-dimensional hidden states, thus obtaining an embedding tensor of dimension (48, 768). Since the output corresponds to 48 embeddings for 1 s of audio, the mean is calculated along the first dimension before applying the sigmoid activation function. The result is an output tensor of dimension (1, 14).

#### 2.6.2. Model Hyperparameters

The following model hyperparameters and their initial set of values are considered.

- Learning rate: The step size at which the optimizer moves towards the minimum of the loss function for each iteration. The initial learning rate is set to 0.00001.
- Optimizer: The optimizer used is AdamW [32] which uses the adaptive gradient algorithm, Adam [33]. It modifies the optimization algorithm of Adam by decoupling weight decay from the optimization steps.
- Number of epochs: The number of training iterations of the complete training dataset. The number of epochs is set to 50.
- Batch size: A batch size of 32 is set, which corresponds to the number of samples loaded during each iteration of the training loop.
- Loss function: BCE loss is used to calculate the loss between the predictions and the target variables. The other alternative is focal loss [34] to deal with an imbalanced dataset.
- Activation function: Sigmoid activation function is used to calculate the output probabilities of each class.

#### 2.6.3. Evaluation of Classifier

For model development, simple computational metrics are used by comparing them with reference annotations. There are two approaches for evaluating AED systems [7];

1. Segment-based evaluation: The system’s output is compared with the reference annotation at a fixed temporal resolution, e.g., 1 s.
2. Event-based evaluation: The system output is compared with the reference annotation in terms of how well the onset/offset times are detected for each sound event. The metric allows for some degree of misalignment of the temporal resolution called collar between the reference and the prediction. A 200 ms collar indicates that a misalignment of 200 ms prediction interval is allowed for the onset and offset times of the reference annotation.

The aforementioned evaluation metrics present two different views of system performance. Segment-based metrics evaluate the temporal activity of each sound event in a given audio, while event-based metrics determine the system’s performance in detecting individual instances of sound [7]. As the proposed AED system can be used to describe an auditory scene, segment-based evaluation suits the application more. Also, since annotations for audio segments of 1 s duration are considered for classification, segment-based metrics are a better choice. However, segment-based metrics have the drawback of overemphasizing the contribution of longer events that span multiple segments [35].

The following metrics are used for the evaluation of the performance of the model [7].

1. Threshold-dependent metrics, Accuracy, F1-Score, Precision, Recall: They are micro-averaging metrics that take class imbalance into consideration by computing the metric on the aggregated values. They are computed for a threshold equal to 0.5.
2. Threshold-independent metrics, mAP and AUC: They are computed with macro averaging, which does not consider class imbalance, as the metric for each class is calculated separately and the average is taken.

## 3. Results

The following factors are considered to evaluate the performance, training, and testing time of the model. Keeping other factors constant, only one set of factors is changed at a time for performance assessment.

- Dataset
- Number of epochs
- Batch size
- Loss Function

### 3.1. Experiments with Different Datasets

The dataset described in Section Exploratory Data Analysis (EDA) is an imbalanced dataset with an imbalance ratio of 17.5. It is referred to as datatset_1 henceforth. It also contains faint sound events or noises, such as driving noises. In addition, no data augmentation is performed on this dataset to train the model. Performance metrics are calculated for the devset dataset during testing the model, which contains test-time-augmented samples [36], in addition to the original samples. The test time augmentation is performed by changing the pitch of each sample is changed by 3, 4, 6, −3, −4, −6 semitones. The evaluation metrics reported are for devsets with test-time augmented data, unless otherwise specified.

In the initial experiment, the model trained on dataset_1 shows a mean accuracy of 0.95 in the devset datasets with a standard deviation of 0.14 across 10 folds. The mean mAP is only 0.39, with a standard deviation of 0.16 across 10 folds. The high accuracy and low mAP can be attributed to the imbalance in the dataset. Events such as driving noise have a higher number of samples with longer duration, and therefore, will contribute more to the ratio between TP and TN while micro-averaging.

**Figure 10:**
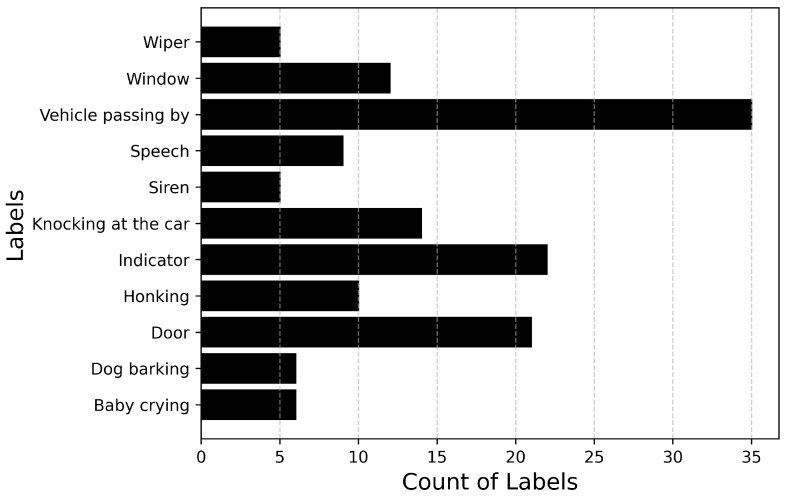
Total number of samples available for each audio event in dataset_2.

**Figure 11:**
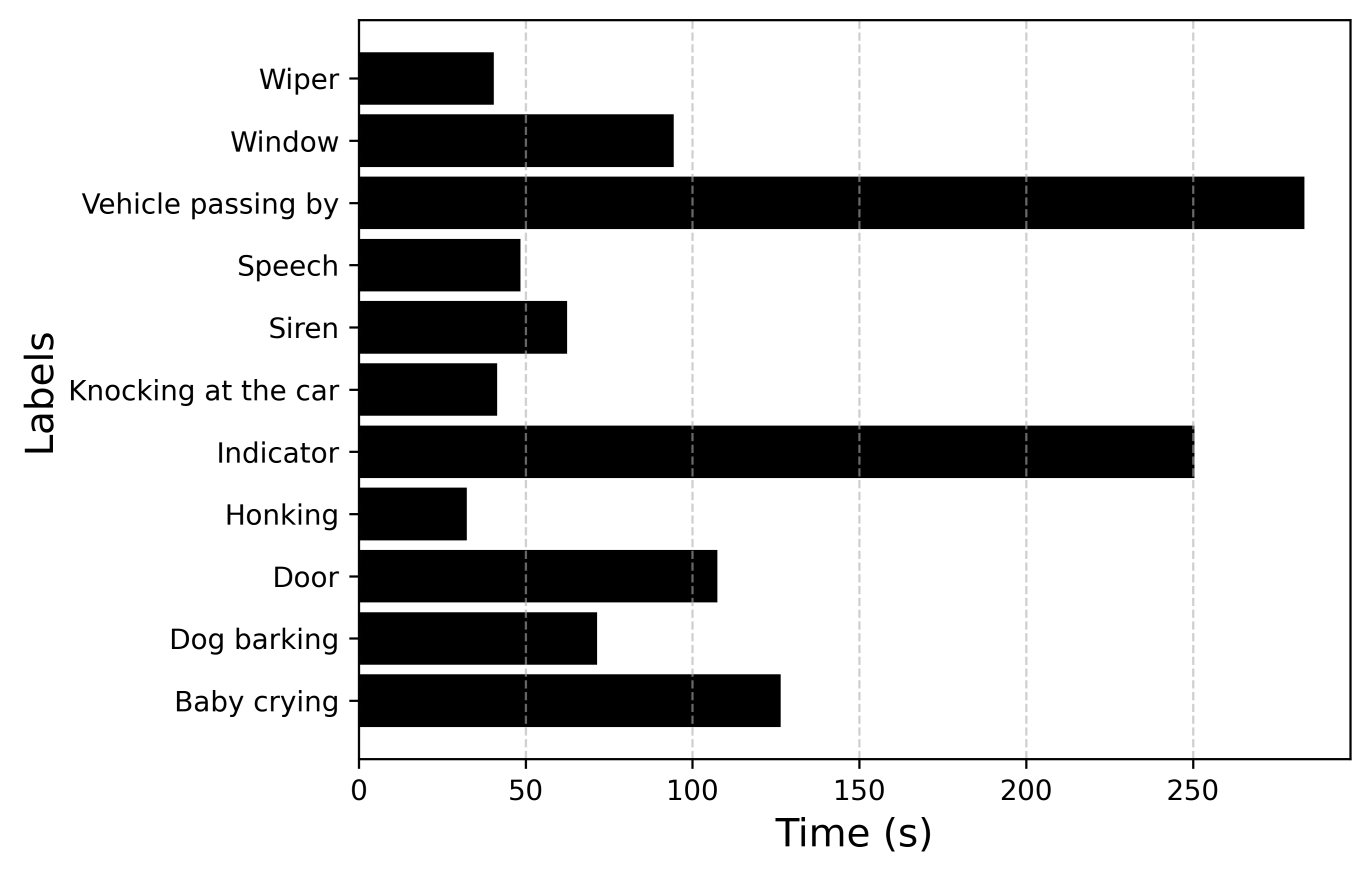
Total duration of each audio event in dataset_2.

Since dataset_1 is heavily imbalanced with a high imbalance ratio, a second dataset, dataset_2 is collected to compare the performance of the model with respect to the dataset_1 on which it is trained. The imbalance is reduced by collecting more training samples from the Internet (Source: Pixabay Sounds (https://pixabay.com/sound-effects/, accessed on 29 April 2024)) and removing the faint sound events: Driving noise, Stationary engine noise, Passenger noise and HVAC. An additional sound event Siren from the defined ontology is collected from the Internet. dataset_2 also contains augmented data in the training and devset datasets for each fold. The pitch of each sample is changed by 3, 4, 6, −3, −4, −6 semitones in both the training and the devset sets for each fold. Figure 10 describes the distribution of the number of samples, and Figure 11 describes the duration of each label in dataset_2. The imbalance ratio of dataset_2 is 5.8 compared to an imbalance ratio of 17.5 of dataset_1. The total number of labels/classes is reduced to 11 from the initial 14 events.

The model trained on dataset_2 shows a mean accuracy of 0.92 in the devset datasets with a standard deviation of 0.16 across 10 folds. The mean mAP is 0.57 with a standard deviation of 0.16 across 10 folds. The accuracy decreased by only a small value of 0.03, while mAP improved by a value of 0.18. Table 2 summarizes the devset accuracy and mAP 10 folds of data for both datasets. It can be inferred from the table that dataset_2 shows better performance even with augmented training and devset data. The results also indicate that the model does not perform well for imbalanced datasets with faint sounds or noise.

### 3.2. Experiments with Different Batch Sizes

The batch size is one of the hyperparameters that can influence the performance of the model. The performance of the model is estimated for batch sizes 128, 64, and 32 using dataset_2, keeping the other parameters unchanged. Table 3 summarizes the results showing the mean accuracy and mAP with the standard deviation.

The results show that a batch size of 32 shows the best results. The performance decreases as the batch size increases. Since the AdamW optimizer uses a dynamic learning rate, the initial learning rate is the same for each batch size. Batch size 128 shows the least deviation of values between folds.

### 3.3. Experiments with Different Loss Functions

Following BEATs, BCE loss is proposed to calculate the loss between the input and the predictions. BCE loss computes the logarithmic value of the probabilities of the target/reference values and predictions. During training, a small subset of training samples called mini-batch is sent to the network to calculate the loss function gradient for training. Therefore, BCE loss is calculated for a mini-batch, resulting in a set of loss values for each sample.

BCE loss $l\left(x,y\right)$ for each sample in a batch of reference value $\left(y_{n}\right)$ and output predictions $\left(x_{n}\right)$ is calculated as follows (https://pytorch.org/docs/stable/generated/torch.nn.BCELoss.html, accessed on 8 April 2024).

(3) $$L=\left\{l_{1},\ldots ,l_{N}\right\},l_{n}=-\alpha_{n}\left[y_{n}\cdot \log x_{n}+\left(1-y_{n}\right)\cdot \log\left(1-x_{n}\right)\right]\;\forall n=1,2,\ldots ,N$$

where *N* is the batch size. $l\left(x,y\right)=mean\left(L\right)$ and $\alpha_{n}$ is a scaling factor applied to each element to balance the loss. If $x_{n}=0$ or $(1-x_{n})=0$, then the corresponding logarithm tends to infinity. According to the solution proposed in the PyTorch documentation for BCELoss, the output of the log function is set to be greater than or equal to −100 so that a finite loss is obtained.

FL as an extension of BCE loss to deal with imbalanced datasets [34]. It adds a factor ${\left(1-x_{n}\right)}^{\gamma }$ to the cross-entropy function. When γ>0, relative loss for samples with the probability of prediction $(x_{n})>0.5$ will be lower, while the loss for samples with $x_{n}<0.5$ will be high. In this way, more focus is placed on samples that are misclassified. Calculation of FL ($FL\left(x_{n}\right)$) and BCE loss ($CE\left(x_{n}\right)$) where $y_{n}=1$ and $\alpha_{n}=1$ is as follows.

(4) $$FL\left(x_{n}\right)={\left(1-x_{n}\right)}^{\gamma }\log\left(x_{n}\right)\;CE\left(x_{n}\right)=-\log\left(x_{n}\right)$$

The performance of the model is evaluated on both BCE and Focal loss functions. The parameter α is set to 1 while γ is set to 2 initially [34]. The results are summarized in Table 4. The results show an improvement in both accuracy and mAP of the devset dataset for 10 folds for the Focal loss function compared to BCE loss.

### 3.4. Model Evaluation on Unseen Data

The experiments discussed so far showed the best results on the devset dataset when the influencing factors are the following.

1. Dataset: dataset_2 shows better results than dataset_1
2. Model Hyperparameter, batch size: A batch size of 32 shows the best performance compared to sized 64 and 128.
3. Model Hyperparameter, loss function: The focal loss function for imbalanced data yields better results than the BCE loss.

Based on the results of the evaluation of the 10 fold devset dataset, the above-mentioned values are obtained. The model is then evaluated on an independent test set that is unseen by the model. That is, a dataset that is not used to tune these parameters should be used to validate the performance of the model. This dataset is henceforth referred to as the unseen dataset. A new data sample for each audio event is collected from the Internet to generate the unseen dataset. The threshold-dependent metrics F1-Score, Precision, Recall, and Accuracy with a threshold equal to 0.5 are considered for evaluation. In addition, the threshold independent metrics mAP, Average precision and ROC are considered. The metrics for each class are obtained to understand the influence of each class on the mean value of F1-Score, accuracy, and mAP. This also helps to understand how well the model can detect each class.

**Figure 12:**
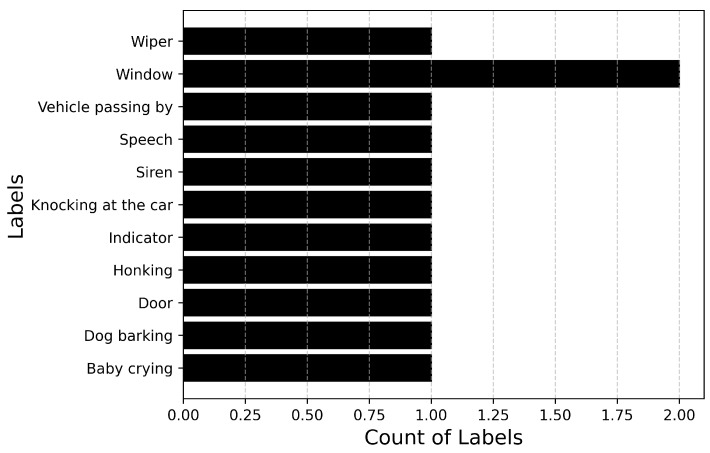
Total number of samples available for each audio event in the unseen dataset.

**Figure 13:**
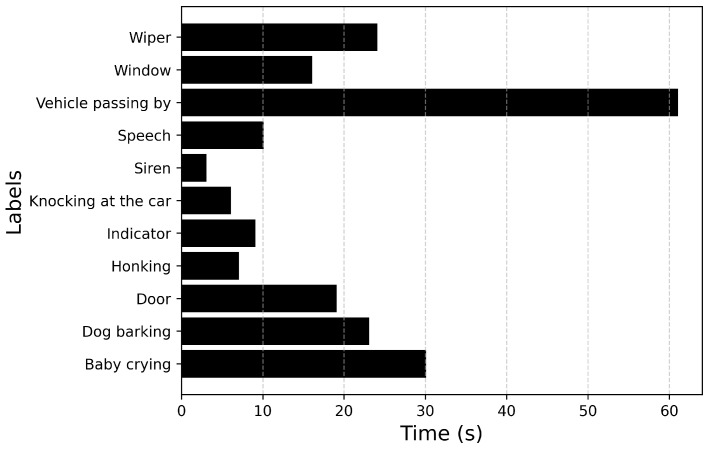
Total duration of each audio event in the unseen dataset.

Figure 12 describes the distribution of the number of samples, and Figure 13 describes the duration of each label. With the updated parameters, the model is trained using the entire dataset_2 (training set and devset), while the model is evaluated on the unseen dataset without test-time augmentation.

**Figure 14:**
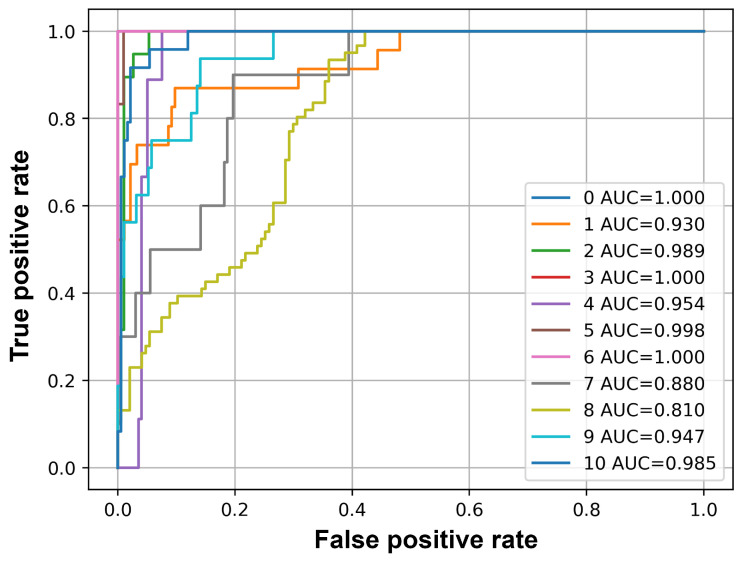
ROC curve and AUC values of each class on unseen data. The AUC number corresponds to the index of each class in the list.

Table 5 lists the values of accuracy, F1-Score, precision, recall, and average precision for each class. Figure 14 plots the ROC curve of each class. The AUC number corresponds to the index of each class in the list.

The results provided in Table 5 suggest that the events Baby crying, Honking and Knocking at the car show good performance according to threshold-dependent metrics; accuracy and F1-Score. The threshold independent value, average precision, shows that in addition to these events, the events Siren, Wiper also show good performance with average precision greater than 0.8. The ROC curves plotted for each class and their corresponding AUC values in Figure 14 show similar results. All the classes have an AUC value greater than 0.8, indicating a good performance of the classifier. However, the events Indicator and Speech have an average precision of less than 0.4.

The mean accuracy, F1-Score, and mAP values are 0.93, 0.39, and 0.77, respectively. The improved mAP value of the model on unseen data may be due to the fact that the data collected from the Internet contain clear and loud audio signals.

### 3.5. Evaluation Results on Augmented Data

**Figure 15:**
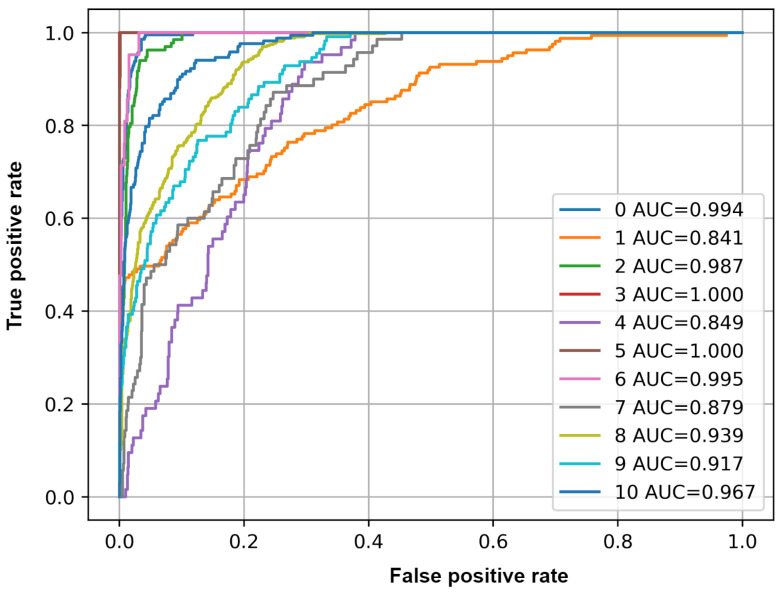
ROC curve and AUC values of each class on unseen data with augmentation. The AUC number corresponds to the index of each class in the list.

The performance of the model is also evaluated after augmenting the unseen data by changing the pitch of each sample 3, 4, 6, −3, −4, −6 semitones, thereby increasing the number of samples to test. This also helps to assess the model performance to pitch variations of the samples. Table 6 lists the values of accuracy, F1-Score, precision, recall, and average precision for each class. Figure 15 plots the ROC curve of each class.

The mean accuracy, F1-Score, and mAP values are 0.93, 0.36, and 0.71, respectively. The performance of the model decreases when the data are augmented. However, the model still shows good performance.

### 3.6. Experiments with Data Containing Overlapping Events

The experiments discussed so far use audio data containing only a single sound event. To understand how well the selected approach distinguishes multiple sound events from a mixture of sounds with varying amplitudes that occur at different instances of time, a dataset generated using ADA is used. Using the augmentation methodology of ADA, synthetic data can be generated that contain multiple sound events with varying amplitudes that overlap each other. The ADA parameters are set to generate sound mixtures of at least two events that completely overlap. The following values are set for ADA parameters.

- Labels: Combination of two labels. There are 11 labels available. A combination of all the labels results in C(11,2)=55 augmented files. This will ensure the overlapping of each event with other events so that there is no bias to any event.
- Frequency of labels: 1 for each label.
- Range of time-shift: The range of (0, 0.1) ensures that the two sound events completely overlap as they begin in this small interval.
- Scaling factor: The amplitude scaling factor is set to a range of (0.2, 0.5). A random value in this range will be applied for scaling.
- Duration: The duration of the augmented audio is set to 20 s. This ensures that longer sound events are not clipped. However, while training and testing the model, only 1 s audio frames corresponding to the start time are fed to the model.

**Figure 16:**
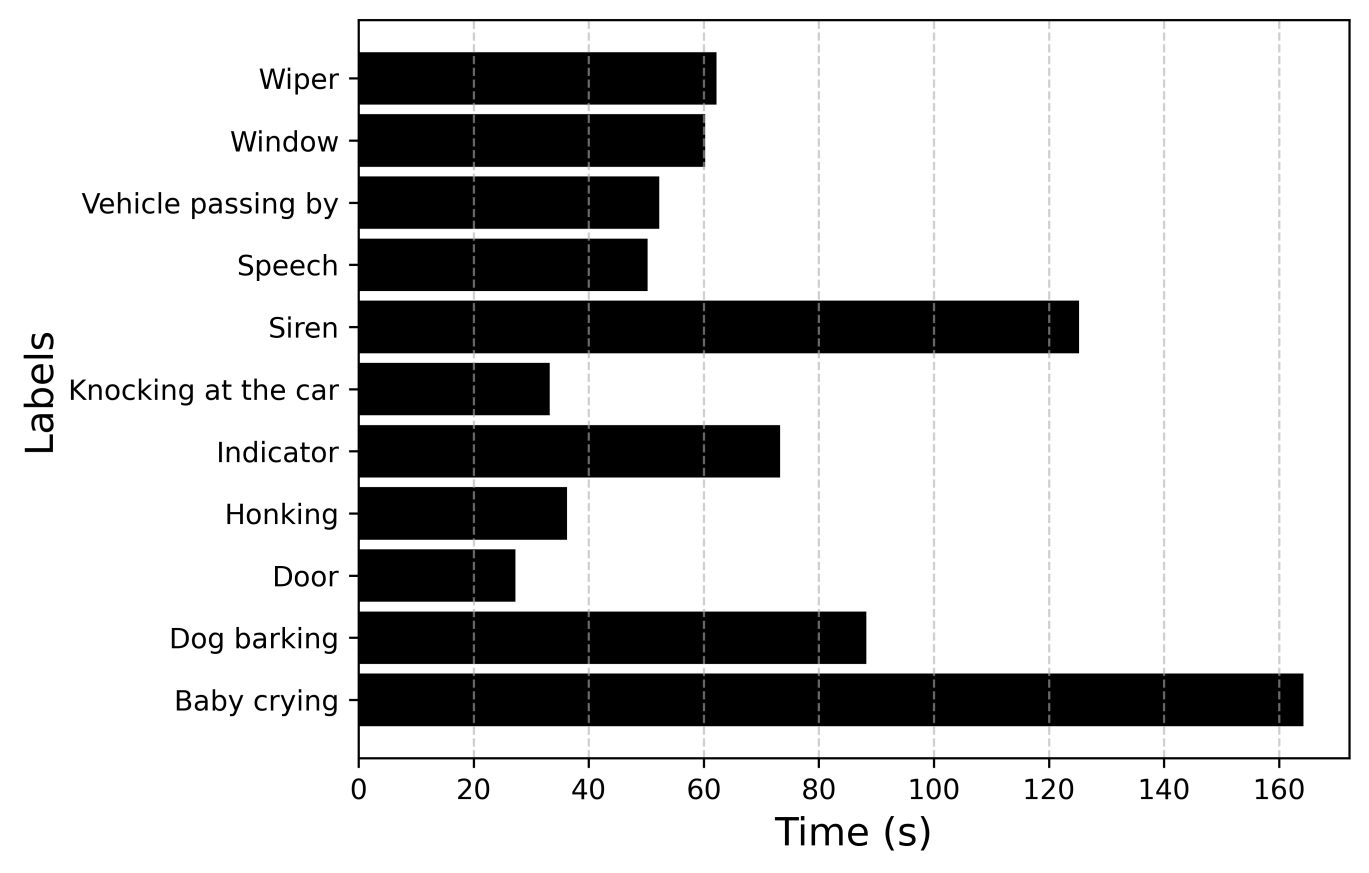
Total duration of each audio event in the dataset generated using ADA.

The training and devset datasets in fold 1 are fed to ADA to generate augmented files. The number of samples will be the same for each label since all combinations of two labels are generated. Figure 16 describes the duration of each label. Data augmentation of changing pitch by the semitones 3, 4, 6, −3, −4, −6 is conducted on each sample for both training and devset sets. The model is developed using only datasets from fold 1.

Since the dataset is balanced, the model is trained and evaluated for both BCE loss and Focal loss functions. Table 7 summarizes the results. The results show that the focal loss performs slightly better than the BCE loss. This can be due to the reason that, though the dataset is balanced, the duration of each sound may have an effect on data balance as 1 s audio frames are fed to train the model.

**Figure 17:**
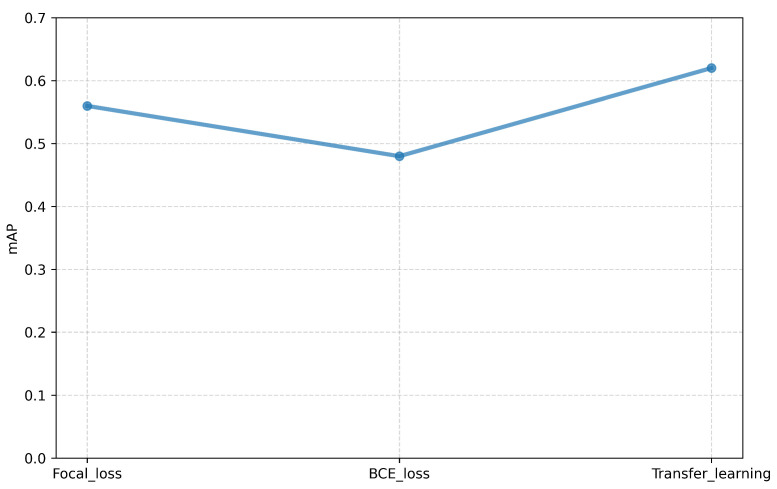
Comparison of mAP on devset data generated using ADA with different loss functions and transfer learning.

The decrease in performance of the model when compared to the model trained on dataset_2 is expected as the data are more complex with multiple overlapping sound events. To understand the effect of transfer learning, the model is initialized with the weights of the model trained on dataset_2. After initializing, the model is trained with the same parameters using the Focal loss function. The experiment shows a small improvement in performance. The accuracy more or less remains the same with a value of 0.88 while mAP increased to a value of 0.62. Figure 17 summarizes the results of the three experiments. From the figure, it can be inferred that initializing the model with a model trained using non-overlapping events can improve the performance.

### 3.7. Model Evaluation on Unseen Data with Overlapping Events

**Figure 18:**
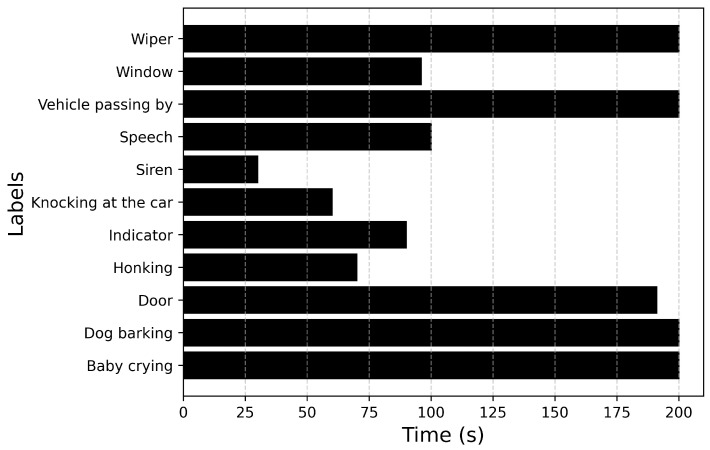
Total duration of each audio event in the unseen dataset generated using ADA.

The unseen dataset (Figure 12 and Figure 13) contains audio files of a single event. This dataset is fed to ADA to generate synthetic data of overlapping events. The same ADA parameters are used. The number of samples for each label will be the same. The duration of each audio event is shown in Figure 18.

**Figure 19:**
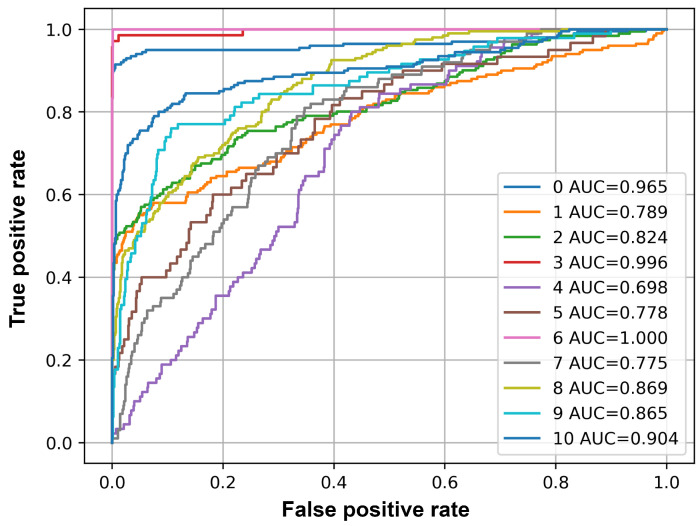
ROC curve and AUC values of each class on unseen data with overlapping events generated using ADA. The AUC number corresponds to the index of each class in the list.

Table 8 summarizes the results of the evaluation for a threshold of 0.5 for the unseen dataset without test-time augmentation. The threshold-dependent metrics for the classes Baby crying and Honking show good results with an F1-Score greater than 0.8. In addition to these classes, the average precision value for the class Siren is close to 1, indicating good performance. It must be noted that the average precision of the class Siren increased from 0.797 to 0.99 when there is an overlap of events. The result suggests that Siren can be detected when there is an overlap of two events. However, this can only be validated with further experiments. Figure 19 plots the ROC curve for each class with their corresponding AUC values. The AUC value is equal to 1.0 for these classes. All classes have a value greater than 0.8, showing good detection performance for all classes.

The mean accuracy, F1-Score and mAP values are 0.89, 0.42, and 0.658, respectively. The accuracy and mAP values decreased, while there was a slight improvement in the F1-Score. Thus, the selected approach indeed performs well when there is an overlapping of events with varying amplitudes. However, this can only be validated with further experiments using varying degrees of overlapping of events.

### 3.8. Evaluation Results on Augmented Data

**Figure 20:**
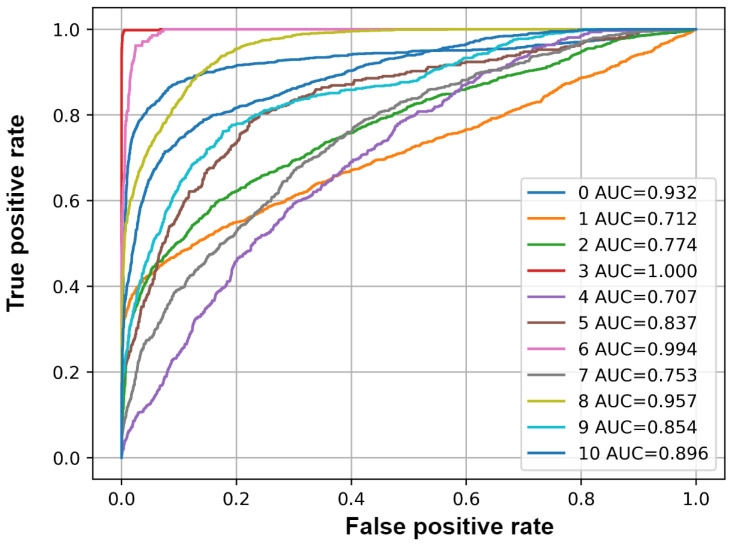
ROC curve and AUC values of each class unseen data generated using ADA, with augmentation. The AUC number corresponds to the index of each class in the list.

The experiment is repeated with the test-time augmented data. Each sample in the dataset is augmented by changing the pitch by 3, 4, 6, −3, −4, −6 semitones. The results with the augmented data are summarized in Table 9. The results show a decrease in the performance of the model for each class. The mean accuracy, F1-Score and mAP values are 0.88, 0.34, and 0.64, respectively. However, the events Baby crying, Honking, Siren show good results. Figure 20 plots the ROC curve and AUC values, suggesting a similar result with an AUC value greater than 0.9 for these classes.

## 4. Discussion

The following sections discuss the overall performance of the model and its limitations.

### 4.1. Model Performance

The following can be concluded from the experiments carried out using segment-based metrics to evaluate the AED model developed.

- The model performs with a mean accuracy, F1-Score, and mAP values of 0.93, 0.39, and 0.77, respectively, for 11 sound events on a test dataset containing only a single event. The threshold-dependent metrics are calculated for a threshold of 0.5.
- The overall performance of the model decreases, with the mean accuracy, the F1-Score and the mAP values equal to 0.89, 0.42, and 0.658, respectively, when the data contains two overlapping events. The threshold-dependent metrics are calculated for a threshold of 0.5. However, the model still shows good performance.
- The sound events Baby crying, Honking and Siren show good results in all experiments. The inference results of one of the unseen data samples show that the model is able to detect the overlapping instances of both Honking and Siren for a threshold of 0.4.

Currently, the model is evaluated on generic evaluation metrics that do not consider the extent of overlapping of sound events. The Polyphonic Sound Detection Score (PSDS) score is a metric that is used in DCASE challenges to evaluate state-of-the-art AED systems, which considers the extent of overlapping of sound events. Although other threshold-independent metrics are used to evaluate the model performance, calculating the PSDS score will help to compare the performance with the state-of-the-art AED systems. In addition, the proposed AED system has been evaluated for a single architecture. Other linear classifiers, such as SVM, can be used in conjunction with BEATs to evaluate performance. Since BEATs has around 90M parameters, the performance of other smaller networks such as BYOL, YAMNet can be explored. Additional experiments can be carried out to improve the performance of the selected model through hyperparameter tuning. Since training time is not optimized, hardware with higher computational capability can be used. If training time is optimized, BEATs model can be fine-tuned to improve performance.

### 4.2. Limitations

- Methods for model calibration can be used to make sure that the predicted probabilities match the real-world probabilities (https://www.tidyverse.org/blog/2022/11/model-calibration/, accessed on 11 May 2024). The predictions of the model are dependent on the defined threshold. During inference, a threshold less than 0.5 is found to predict events with more accuracy. Tuning the threshold to an optimum value can improve the classifier prediction during inference. The model can also be trained to predict parts of audio containing no sound as Silence.
- Since the model is trained using a small dataset with a limited number of samples for each class, some classes show poor performance. Sound events, such as Indicator and Speech, have an average precision of less than 0.4. This can also be attributed to the fact that these sound events are more of a continuous nature. The performance of other classes can still be improved through hyperparameter tuning.
- The performance of the model for overlapping sound events is evaluated for only a set of combinations of sound events that overlap. Depending on the extent of overlap and the number of overlapping events, the performance of the model may vary.

## 5. Conclusions

The paper introduces an AED system that can detect various sound events within the vicinity of a vehicle. Though there is active research in the area of sound event detection methodologies, they mainly focus on environmental sounds. There is also a scarcity of datasets when it comes to sound data in an automotive context. The approach described in the paper tackles this challenge by collecting real recordings of sound events according to the proposed ontology. A novel augmentation methodology is applied in order to generate polyphonic sound mixtures from these recordings. Using the pretrained model BEATs as a frozen feature extractor and a single-layer neural network for classification, the proposed system is able to predict audio events every 1 s in order to create an audio scene description. The results of the experiments carried out suggest that the model performs relatively well on the unseen datasets. Given sufficient data, a similar approach can be applied to a broader context of audio applications in the automotive domain.

Currently, the proposed system detects sound events in audio files stored locally. Therefore, future work can constitute deploying the AED model into an embedded system, where the system uses microphones to capture audio while the deployed model predicts the sound events. Since the prediction occurs every 1 s, a ring buffer of 1 s can be designed where audio events in the incoming 1 s duration are predicted, then for the next 1 s, and so on continuously in real-time. In this way, the model can be trained with large amounts of data if the system is installed in cars. In order to account for the complexity of the BEATs model, optimization techniques such as Low-Rank Adaptation (LoRA) [37] can be used for deploying the model into resource constraint embedded systems. Alternatively, models such as YAMNet and BYOL with fewer parameters can also be explored. Integrating methods such as federated learning can protect the privacy of car users while collecting data from different cars.

Further experiments must be carried out in order to explore the model’s robustness to more real-world conditions. For further investigation, synthetic data from the ADA software can be generated that contain more than two overlapping events. However, the real-world automotive environment is more complex, and further extensive studies must be performed to evaluate the model’s performance. Furthermore, self-supervised learning strategies can be introduced to replace the tedious process of collecting annotated data. Currently, the audio scene description is created with the predictions for every 1 s. This can be further extended with an LLM model to create a natural language description of the audio scene. Models like SALMONN [38], LTU [39] show a similar approach.

## Figures and Tables

**Table 1:** Comparison of different pre-trained audio networks on AudioSet dataset.

Model	Performance (mAP)	Complexity (Million)	License
YAMNet [21]	0.389	3.7M	Apache License 2.0
PANNs [22]	0.439	81M	MIT License
AST [23]	0.459	86M	BSD 3-Clause License
PaSST [24]	0.471	86M	Apache License
BEATs [20]	0.486	90M	MIT License

**Table 2:** Summary of results of experiments with different datasets. Mean accuracy, mAP with the respective standard deviation along with 95% confidence intervals for 10-folds, are calculated.

Dataset	Accuracy	mAP
dataset_1	0.95 ± 0.014, (0.940, 0.960)	0.39 ± 0.16, (0.276, 0.504)
dataset_2	0.92 ± 0.016, (0.909, 0.931)	0.57 ± 0.16, (0.456, 0.684)

**Table 3:** Summary of results of experiments with different batch sizes. Mean accuracy, mAP with the respective standard deviation along with 95% confidence intervals for 10-folds are calculated.

Batch Size	Accuracy	mAP
32	0.92 ± 0.02, (0.906, 0.934)	0.55 ± 0.15, (0.443, 0.657)
64	0.9 ± 0.006, (0.896, 0.904)	0.4 ± 0.08, (0.343, 0.457)
128	0.91 ± 0.008, (0.904, 0.916)	0.37 ± 0.07, (0.320, 0.420)

**Table 4:** Summary of results of experiments with different loss functions. Mean accuracy, mAP with the respective standard deviation along with 95% confidence intervals for 10-folds, are calculated.

Loss Function	Accuracy	mAP
BCE loss	0.92 ± 0.02, (0.906, 0.934)	0.55 ± 0.15, (0.443, 0.657)
Focal loss	0.93 ± 0.02, (0.916, 0.944)	0.64 ± 0.14, (0.540, 0.740)

**Table 5:** Evaluation metrics for each class on unseen data.

Index	Label	Accuracy	F1-Score	Recall	Precision	Avg_Precision
0	Baby crying	0.99	0.967	1.0	0.937	1.0
1	Dog barking	0.937	0.606	0.434	1.0	0.793
2	Door	0.947	0.645	0.526	0.833	0.843
3	Honking	0.99	0.833	0.714	1.0	1.0
4	Indicator	0.937	0.0	0.0	0.0	0.33
5	Knocking at the car	0.99	0.8	0.666	1.0	0.958
6	Siren	0.985	0.0	0.0	0.0	1.0
7	Speech	0.951	0.0	0.0	0.0	0.394
8	Vehicle passing by	0.72	0.093	0.049	1.0	0.622
9	Window	0.937	0.38	0.25	0.8	0.684
10	Wiper	0.886	0.0	0.0	0.0	0.866

**Table 6:** Evaluation metrics for each class on unseen data with augmentation.

Index	Label	Accuracy	F1-Score	Recall	Precision	Avg_Precision
0	Baby crying	0.964	0.888	0.966	0.821	0.961
1	Dog barking	0.934	0.599	0.441	0.934	0.624
2	Door	0.938	0.524	0.368	0.907	0.851
3	Honking	0.984	0.71	0.551	1.0	1.0
4	Indicator	0.952	0.0	0.0	0.0	0.0
5	Knocking at the car	0.99	0.8	0.666	1.0	0.994
6	Siren	0.985	0.0	0.0	0.0	0.797
7	Speech	0.951	0.0	0.0	0.0	0.261
8	Vehicle passing by	0.738	0.201	0.112	0.96	0.849
9	Window	0.935	0.287	0.169	0.95	0.576
10	Wiper	0.884	0.0	0.0	0.0	0.813

**Table 7:** Summary of results of experiments with different loss functions for dataset from ADA for a single fold.

Loss Function	Accuracy	mAP
BCE loss	0.86	0.48
Focal loss	0.87	0.56

**Table 8:** Evaluation metrics for each class on unseen data with overlapping events generated using ADA.

Index	Label	Accuracy	F1-Score	Recall	Precision	Avg_Precision
0	Baby crying	0.947	0.884	0.94	0.835	0.960
1	Dog barking	0.87	0.561	0.39	1.0	0.696
2	Door	0.869	0.525	0.356	1.0	0.721
3	Honking	0.984	0.88	0.785	1.0	0.987
4	Indicator	0.878	0.109	0.07	0.184	0.178
5	Knocking at the car	0.920	0.336	0.316	0.385	0.342
6	Siren	0.982	0.636	0.466	1.0	0.994
7	Speech	0.893	0.0	0.0	0.0	0.275
8	Vehicle passing by	0.795	0.0769	0.040	1.0	0.712
9	Window	0.913	0.456	0.354	0.641	0.535
10	Wiper	0.808	0.181	0.1	1.0	0.842

**Table 9:** Evaluation metrics for each class on unseen data generated using ADA, with augmentation.

Index	Label	Accuracy	F1-Score	Recall	Precision	Avg_Precision
0	Baby crying	0.906	0.797	0.861	0.741	0.882
1	Dog barking	0.846	0.534	0.415	0.75	0.599
2	Door	0.818	0.212	0.120	0.899	0.591
3	Honking	0.976	0.809	0.679	1.0	0.997
4	Indicator	0.900	0.081	0.046	0.353	0.416
5	Knocking at the car	0.933	0.343	0.271	0.469	0.416
6	Siren	0.972	0.25	0.142	1.0	0.871
7	Speech	0.893	0.0	0.0	0.0	0.331
8	Vehicle passing by	0.804	0.148	0.080	1.0	0.877
9	Window	0.912	0.461	0.369	0.616	0.543
10	Wiper	0.80	0.11	0.062	1.0	0.784

## Data Availability

Data available on request due to restrictions. The recordings and the link to the public datasets are available by request to the corresponding author.

## Abbreviations

The following abbreviations are used in this manuscript:

A2B	Automotive Audio Bus
ADAS	Advanced Driver Assistance Systems
ADA	Audio Data Augmentation System
AED	Acoustic Event Detection
AST	Audio Spectrogram Transformer
AUC	Area Under the ROC curve
BCE	Binary Cross Entropy
BEATs	Bidirectional Encoder representation from Audio Transformers
BLSTM	Bidirectional Long Short Term Memory
BYOL	Bootstrap Your Own Latent
CNN	Convolutional Neural Network
CRNN	Convolutional Recurrent Neural Network
DCASE	Detection of Acoustic Scenes and Events
DNN	Deep Neural Networks
FNN	Feed Forward Neural Network
GMM	Gaussian Mixture Model
GRU	Gated Recurrent Unit
HMM	Hidden Markov Model
HVAC	Heating, Ventilation, and Air Conditioning
LiDAR	Light Detection and Ranging
LLM	Large Language Model
mAP	mean Average Precision
MFCC	Mel Frequency Cepstral Coefficients
NMF	Nonnegative Matrix Factorization
PaSST	Patchout faSt Spectrogram Transformer
PSDS	Polyphonic Sound Detection Score
RADAR	Radio Detecting and Ranging
RNN	Recurrent Neural Networks
ROC	Receiver Operating Characteristic
SVM	Support Vector Machine
YAMNet	Yet Another Music Recognition Network
